# Deep learning and machine learning in psychiatry: a survey of current progress in depression detection, diagnosis and treatment

**DOI:** 10.1186/s40708-023-00188-6

**Published:** 2023-04-24

**Authors:** Matthew Squires, Xiaohui Tao, Soman Elangovan, Raj Gururajan, Xujuan Zhou, U Rajendra Acharya, Yuefeng Li

**Affiliations:** 1 School of Mathematics, Physics and Computing, University of Southern Queensland, Toowoomba, QLD Australia; 2 Belmont Private Hospital, QLD Brisbane, Australia; 3 School of Business, University of Southern Queensland, Springfield, QLD Australia; 4 School of Computer Science, Queensland University of Technology, Brisbane, QLD Australia

**Keywords:** Psychiatry, Artificial intelligence, Depression, Deep learning, Neural networks, Treatment response prediction

## Abstract

Informatics paradigms for brain and mental health research have seen significant advances in recent years. These developments can largely be attributed to the emergence of new technologies such as machine learning, deep learning, and artificial intelligence. Data-driven methods have the potential to support mental health care by providing more precise and personalised approaches to detection, diagnosis, and treatment of depression. In particular, precision psychiatry is an emerging field that utilises advanced computational techniques to achieve a more individualised approach to mental health care. This survey provides an overview of the ways in which artificial intelligence is currently being used to support precision psychiatry. Advanced algorithms are being used to support all phases of the treatment cycle. These systems have the potential to identify individuals suffering from mental health conditions, allowing them to receive the care they need and tailor treatments to individual patients who are mostly to benefit. Additionally, unsupervised learning techniques are breaking down existing discrete diagnostic categories and highlighting the vast disease heterogeneity observed within depression diagnoses. Artificial intelligence also provides the opportunity to shift towards evidence-based treatment prescription, moving away from existing methods based on group averages. However, our analysis suggests there are several limitations currently inhibiting the progress of data-driven paradigms in care. Significantly, none of the surveyed articles demonstrate empirically improved patient outcomes over existing methods. Furthermore, greater consideration needs to be given to uncertainty quantification, model validation, constructing interdisciplinary teams of researchers, improved access to diverse data and standardised definitions within the field. Empirical validation of computer algorithms via randomised control trials which demonstrate measurable improvement to patient outcomes are the next step in progressing models to clinical implementation.

## Introduction

Conditions associated with poor mental health place a significant burden on the Australian health care system. Some evidence [1, 2] suggests despite government investment, availability of inpatient mental health services sits below the level of demand. Additionally, demand for mental health services is expected to grow further as the psychological effects of the Coronavirus pandemic are felt by the population [3]. To support increases in demand, modern algorithms have the potential to streamline the diagnosis of mental health conditions and support the improved targeting of treatments utilising a data-driven paradigm.

Advanced computing techniques including machine learning, deep learning and artificial intelligence are well positioned to positively contribute to mental health outcomes of individuals [4]. With these advanced techniques comes the potential for precision medicine. The aim of precision medicine is to tailor treatments to the individual patient as opposed to population averages [5]. More recently, the notion of precision medicine has opened the possibility of personalised mental health care. This personalisation is often referred to as precision psychiatry. Research exploring the ways artificial intelligence, machine learning and big data can be used to support mental health treatment is growing rapidly. Evidence of this growth is demonstrated by Brunn et al. [6] who observed a 250% increase in publications exploring artificial intelligence and psychiatry between 2015 and 2019 on PubMed.

Artificial intelligence will be a part of mental health care in the future. This notion is widely acknowledged by practising psychiatrists [7]. Doraiswamy et al. [7] reported results from a global survey of psychiatrists in which most acknowledge artificial intelligence will impact the future of their profession. However, clinicians vary on the degree of disruption artificial intelligence will have on the field. Few psychiatrists believe artificial intelligence will be able to “provide empathetic care to patients” [7, p. 3]. However, a slim majority believe artificial intelligence will be able to diagnose or predict patient outcomes “better than the average psychiatrist” [7, p. 4]. Whilst opinion differs on the level of artificial intelligence disruption, most clinicians believe that artificial intelligence will never completely replace mental health professionals [8, 9].

While artificial intelligence may never replace the personalised, empathetic care that a psychiatrist can provide, this paper will detail the data-driven informatics approaches positioned to revolutionise the diagnosis, detection and treatment of depression.

Pattern recognition is one of the key strengths of machine and deep learning algorithms. These techniques have shown some promise in identifying generalisable patterns amongst patients suffering mental health conditions. For example, Carrillo et al. [10] demonstrated a Gaussian Naive Bayes classifier using transcribed textual data could successfully categorise healthy controls from patients suffering depression with a *F*1-score of 0.82. Given the observed difficulty in diagnosing mental health conditions, systems with the ability to diagnose depression provide some benefit to Psychiatrists. Compared to other domains of medicine, mental health conditions have no objective markers of disease [11]. This lack of objective marker is one of several key diagnostic challenges in identifying psychopathology [12]. Current diagnostic systems are being questioned due to the significant heterogeneity of symptoms amongst populations diagnosed with the same condition [13]. Unsupervised learning techniques are supporting the identification of distinct subtypes of depression or potentially new diagnosis. Exploring depression heterogeneity, Drysdale et al. [11] used an unsupervised learning technique, hierarchical clustering, to explore functional connectivity amongst patients diagnosed with depression. While the majority of research surveyed in this paper utilises supervised techniques, unsupervised techniques provide researchers with the opportunity to uncover previously unknown relationships. The work by Drysdale et al. [11] uncovered four distinct biotypes of depression based on fMRI scans. Each of these biotypes was shown to respond differently to rTMS treatment. Given each subtype responded differently to treatments it is possible that each subtype represents a unique condition. This work highlights the possibility of artificial intelligence systems to support a transition to new diagnostic taxonomies.

As well as supporting the detection and diagnosis of mental health conditions, modern computing techniques offer the potential to personalise treatment prescription. Currently, clinicians rely on a trial and error approach to find the best antidepressant for a patient [4, 14, 15]. However, groundbreaking research by Chang et al. [16] demonstrates the potential for psychiatrists to evaluate the likely effect of an antidepressant drug before prescribing it. Their work shows using an artificial neural network, the Antidepressant Response Prediction Network, or ARPNet, can reliably predict the effect of an antidepressant prior to treatment. These technologies raise the possibility of treatment tailored to the patient level.

In its earliest form, artificial intelligence aimed to synthetically reproduce human processes [17]. In its infancy, symbolic artificial intelligence was the aim of such research. The goal of symbolic artificial intelligence work was to “carry out a series of logic-like reasoning steps over language like representations” [18, p. 17]. However, symbolic artificial intelligence is no longer the predominant area of interest for the majority of artificial intelligence researchers. Instead, pattern recognition through the use of artificial neural networks now dominates the field [17]. The seminal work of Rosenblatt [19] provides the first example of the perceptron, the foundation of much of the current work on neural networks. Increasingly, with advances in technology, these networks have become larger leading to the advent of deep learning [20]. The depth, in deep learning refers to the number of hidden layers in an artificial neural network. However, no agreed-upon definition exists to what constitutes a ‘deep’ neural network [20, 21]. Sheu [22] assert a deep neural network has a minimum of 3 layers, an input layer, a hidden layer and an output layer. However, in general, modern researchers require several hidden layers before declaring a network a deep neural network.

**Fig. 1:**
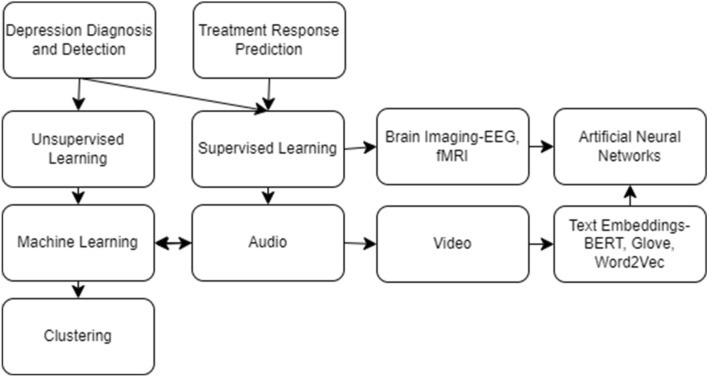
Content map

In this paper, we will define artificial intelligence as the broad field of techniques, encompassing all of machine learning, the neural network and deep learning. In turn, machine learning will be used to refer to all non-neural network techniques, regardless of depth. This will include techniques such as linear regression, logistic regression and nearest neighbours. Given the ambiguity in the difference between artificial neural networks and deep learning, the terms will be used somewhat interchangeably. Additionally, to help the reader navigate this paper we have an included a concept map in Fig. 1. This figure provides a high-level representation of the data types and techniques being used to explore the field of depression detection, diagnosis and treatment response prediction.

This paper explores the ways in which modern phenomenons such as machine learning and deep learning are contributing to improvements in the detection, diagnosis and treatment of mental health condition. As such, this article contributes:

- An overview of the current data types and methodologies being used by the research community to progress the detection, diagnosis and treatment response prediction of mental health conditions.
- A survey of the modern computational techniques used for the detection, diagnosis and treatment response prediction of mental health conditions. Including software repositories useful for feature generation.
- A summary of the current methodological and technical limitations facing the field researching precision psychiatry.
- Reflection on the current issues facing the field and possible solutions to guide future research.

Currently, detection systems are the most widely researched areas utilising artificial intelligence to support mental health care. Section 2 provides an overview of the ways modern computational techniques are shaping the detection of mental health conditions. This area of study focuses on the design of systems built using multimodal data, such as audio, video and text data to detect mental health conditions. Section 2.3 provides a summary of the modern systems being used to revolutionise current diagnostic systems, including the vast heterogeneity within current diagnostic categories. Additionally, Sect. 3 provides an in-depth overview of one of the more recent advances in the literature, treatment response prediction. To date, detection models for mental illness have dominated the literature. More recently, using data to predict how effective a treatment might be has become an exciting area of research with much potential.

## Informatics paradigms and the diagnosis and detection of depression

Traditionally the study of psychiatry has relied heavily on statistical inference. Inferential statistics are mainly concerned with underlying distributions. Inference “creates a mathematical model of the data generation process to formalize understanding or test a hypothesis about how a system behaves” [23, p. 233]. Where statistical inference focuses on explaining group differences based on a handful of variables. Prediction is instead suited to larger variable sets to make predictions around some target variable. Machine learning is interested in prediction and pattern recognition. Diagnosing a mental health condition requires recognising common patterns associated with a condition to make a prediction at an individual level. More recently, advances in computing processing power have led to the rise of deep learning models.

### Machine learning to support the diagnosis of depression

Depression detection using machine learning has grown quickly, taking advantage of the vast corpus of text generated by social media. The diagnosis of depression from social media data can be understood as a supervised learning task where posts are labelled as depression or not depression. From the literature surveyed two classes of experiments emerge; Research where depression status is confirmed by psychometric test or clinical opinion and research relying on self-report.

When building depression detection systems variables must be preprocessed for model input. Preparing text for machine learning is referred to as Natural Language Processing (NLP). NLP is the process of converting natural language to numerical representations that are computer interpretable. Observed processing techniques within the literature are the LIWC [24], Affective Norms for English Words [25], LabMT [26], Latent Dirichlet Allocation [27], *n*-grams and bag-of-words [28, see Chapter 3]. *N*-grams and bag-of-words are elementary methods to numerically represent text, where bag-of-words is a simple text representation which counts the frequency of each word within a text document [28]. Despite their simplicity, the utility of these methods has been shown on several occasions [29–33]. More recently, audio and visual features have been included with several systems utilising processed audio features [34–36] and others which combine audio and visual information [37, 38].

Text data have become a staple feature of most depression detection systems. In pioneering work, De Choudhury et al. [39] attempted to predict depression in Twitter users. Similarly, Reece et al. [31] sought to use Twitter content to classify depressed users. Both [31, 39] recruited participants via crowdsourcing and validated a depression diagnosis using psychological diagnostic questionnaire. For example, in both [31, 39] participants completed the Center for Epidemiological Studies-Depression (CES-D; [40]) self-report survey. Results from this diagnostic tool were used as the ground truth labels between depressed and non-depressed individuals. In these examples [31, 39] researchers used surveys to attempt to confirm a depression diagnosis, however, some works rely on self reported depression status without survey data. De Choudhury et al. [39] developed one of the earliest depression diagnosis systems in the literature. Motivated by the limitations of self-report questionnaires De Choudhury et al. [39] aimed to construct an objective depression measurement. These early text analysis systems exploring word usage and depression relied on dictionary-based text analysis software. These systems used hard-coded dictionaries of words selected and grouped by their psychometric properties. Primarily used by clinicians these systems sought to explore differences in language use between depressed and non-depressed individuals.

The Linguistic Inquiry and Word Count (LIWC; [24]) was one of the earliest examples of a text analysis software. Before the LIWC, text analysis was generally conducted by human raters, however, this was inefficient, costly, and emotionally draining for judges [41]. Furthermore, raters rarely agreed when evaluating the same piece of writing [41]. Hence, computational solutions provide a faster and more consistent alternative. For depression researchers the LIWC allowed the comparison of language usage between depressed and non-depressed populations. Combining linguistic features, such as the LIWC, with Twitter behavioural data, De Choudhury et al. [39] showed a support vector machines (SVM) classifier could predict a depressive episode up to twelve months in advance. Similarly, in the Japanese context Tsugawa et al. [33] combined linguistic features with users’ Twitter information to detect depression on Twitter. Along with analysing the sentiment of posts, Tsugawa et al. [33] show understanding the underlying topics of tweets to be helpful in distinguishing depression status. Combining LDA, a statistical technique used to identify underlying topics within a passage of text [27], with sentiment and twitter data Tsugawa et al. [33] returned an *F*1-score of 0.46. Both [39, 33] these works used questionnaires to validate depression status. In contrast, Hassan et al. [30] used self-reported depression status to generate a text corpus. Using SVM and multiple linguistic features, Hassan et al. [30] achieved a *F*-score of 0.81 in their depression measurement system. The LabMT and ANEW could be broadly described as classes of sentiment analysers. These dictionaries associate each word with a valence which can be then input into a machine learning classifier. The LabMT word list contains 5000 of the most common words used on popular online platforms such as Twitter [26]. Similarly, The ANEW is a dictionary of words and an associated valence [25]. Furthermore, these tools can be manipulated to a research problem. For example, Shen et al. [42], constructed the Valence, Arousal and Dominance (VAD) tool from the ANEW. Shen et al. [42] assert their VAD tool was useful for explaining human emotions within text documents.

Reece et al. [31] used a random forest classifier to detect depression indicators in a Twitter corpus. Similar to methods described previously, a depression diagnosis was verified using psychological questionnaire. Reporting a *F*1-score of 0.644 Reece et al. [31] assert their work offers strong support for a computational method to detect depression. Similarly, Islam et al. [43] found all LIWC dimensions fed into a KNN showed promise in the detection of depression. Table 1 provides a summary of the classification systems identified under the scope of this survey. However, this table does not include deep learning algorithms or neural networks which are discussed in Sect. 2.2.

Some detection systems base their ground truth labels on the self reported health status of the participant. All of Pirina and Çöltekin [44], Islam et al. [43], Tadesse et al. [32], Shen et al. [42] rely on self-report of depression status. These works used pattern matching to identify depression indicative content, searching for that include sentences like, “I have depression.” Depression indicative posts are labelled and used as training data for supervised learning techniques. Unfortunately, when datasets are developed in this manner depression status is never assessed by psychologist or questionnaire. As such, some mislabeled examples must be expected within the dataset [44]. Despite these limitations, large datasets allow researcher to uncover algorithms and feature sets which can be applied to the detection and diagnosis of depression.

**Table 1:** Detection systems and their features

Researcher	Method	Features	Dataset	*F*1-score
McGinnis et al. [35]	Logistic regression and linear SVM	Zero crossing rate, Mel frequency cepstral coefficients and the *Z*-score of the power spectral density	McGinnis et al. [35]	–
Tadesse et al. [32]	SVM	LIWC, LDA and Bigram	Pirina and Çöltekin [44]	0.91
Islam et al. [43]	Coarse KNN	LIWC	Islam et al. [43]	0.71
Reece et al. [31]	Random Forest	LIWC, LabMT, ANEW and Unigram	Reece et al. [31]	0.61
Hassan et al. [30]	SVM	*N*-gram, POS tagger, Sentiment Analyser and Negation	Hassan et al. [30]	0.81
Shen et al. [42]	Multimodal dictionary learning	LIWC, VAD, LDA, word2vec and Twitter behaviour data	Shen et al. [42]	~ 0.85
Deshpande and Rao [29]	Multinominal Naive Bayes	Bag-of-words	Deshpande and Rao [29]	0.83
Tsugawa et al. [33]	SVM	Bag-of-words, LDA, sentiment analysis+user specific information	Tsugawa et al. [33]	0.46
De Choudhury et a.l [39]	SVM	ANEW,LIWC and Twitter behaviour data	De Choudhury et al. [39]	0.68

The relationship between mental health status and speech is well established [45]. While text features focus on the content of speech, audio features involve the processing of the sound to analyse a variety of measurements. The inclusion of audio features in depression detection systems requires signal processing of the audio for it to be included in classification models. Several open source speech processing repositories exist and are used in the literature including COVAREP [46], openSMILE [47] to aid in feature extraction. Equivalent tools for processing of visual data technologies include measurements such as Facial Action Units (FAU) [37, 38]. Where FAU’s “objectively describe facial muscle activations” [48, p. 2].

From Table 1, we see distinct performance difference depending on how depression status was validated. These findings raise concerns around how accurate methods relying on self-report actually are. Existing methods fail to capture this uncertainty inherent within self-reported data. Mental health data is often subjective which makes creating establishing ground truth labels more difficult. Future work should endeavour to adopt emerging data science techniques such as Bayesian Neural Networks (BNN) which are currently being explored to account for inherent data uncertainty.

### Artificial neural networks and deep learning: from hand-crafted features to text embeddings and beyond

To date, the tools described above have shown to be efficacious in the development of depression detection system. For machine learning, feature selection is a vital part of model building. However, the development of these features can be laborious and time consuming [49]. As such, recent approaches have sought to automate the feature selection process. One of the strengths of deep learning algorithms is their ability to learn feature representations without the need for lengthy feature selection process.

More recently, deep learning has been applied to the detection of depression from text, audio and visual features. Similar to the machine learning techniques discussed in Sect. 2.1, deep learning methods are trained using labelled examples to discern patterns between individuals with and without depression. In contrast to traditional machine learning techniques, in general deep learning algorithms do not require hand-crafted features. Advanced deep learning algorithms that use textual data require word embeddings to make text machine readable. These embeddings are vector representations of text documents [28]. Deep learning algorithms use these vector representations to then learn features from the provided data [49]. Neural word embeddings such as Word2Vec [50], Global Vectors for Word Representation [51, GloVE] and more recently transformer based architectures such as Google’s Bidirectional Encoder Representation from Transformers [52, BERT] are becoming far more prevalent in depression research for representing text numerically for deep learning models.

To date, little work has applied deep learning to the assessment of psychopathology [53]. There are likely several reasons for the delay in adoption of these techniques. One of which is concerns around the lack of transparency in how deep learning models make their predictions. These concerns have led some [54] to argue against the use of deep learning models for important health-related decisions. Instead preferencing traditional techniques which have greater prediction transparency. Despite concerns about model transparency, deep learning models have been shown to significantly outperform traditional machine learning techniques for the detection of depression. Cong et al. [49] proposed a system which combined XGBoost with an Attentional Bidirectional LSTM (BiLSTM). Their work was tested on the Reddit Self-Reported Depression Dataset (RSDD; [55]). Compared against several systems applied to the same dataset (including an SVM using LIWC features), the authors [49] reported a *F*1-score of 0.60. Despite its performance, previous sections have outlined some issues with self report data (see Sect. 2.1). While the system design may be useful, a dataset trained on a self-reported sample may not be applicable in a clinical setting. Rosa et al. [53] developed a deep learning approach for the recognition of stressed and depressed users. Their work used a dataset constructed using 27,308 labelled Facebook messages. The authors assert their Convolutional Neural Network (CNN) BiLSTM-Recurrent Neural Network (RNN) using SoftMax recorded the best results for recognising depressed users. They [53] reported an *F*1-score of 0.92 with a precision of 0.9 for the recognition of depressed users, significantly outperforming a Random Forest and Naive Bayes. However, it is not clear from their paper how responses were labelled or participants recruited. As highlighted in previous sections how study participants are recruited has a huge impact on model performance.

As such, textual data are commonly used data type for detection of mental health conditions. Building upon the success of text-based systems emerging research is utilising multimodal data to detect depression. The Distress Analysis Interview Corpus (DAIC; [56]) is a database of 621 interviews collected utilising a combination of face to face, teleconference and automated agent interview. The dataset includes text, physiological data (such as electrocardiogram), voice recordings and psychological questionnaire scores. Utilising this dataset, Alhanai et al. [34] combined audio with transcribed transcripts to predict depression categorically using a neural network. Their approach trained two LSTM models separately, one trained on audio features, the other using text features. Each model was trained individually, with their own weights and hyperparameter. The outputs of these two separate models were then concatenated and passed to another LSTM layer. The best performing model reported by Alhanai et al. [34] utilised both text and audio features to report a *F*1-score of 0.77. Highlighting the benefits of combining multiple data types in model performance.

Chen et al. [57] applied a deep learning approach to automate the diagnosis of perinatal depression. Their method used WeChat, a popular social media application, in the design of their system. Participants were recruited from doctors based on their Edinburgh Postnatal Depression Score (EDPS). Their work [57] was built using Long Short Term Memory (LSTM), a type of neural network. In this work the authors assert their findings match the findings of the EDPS in their sample however, little evidence is offered to support this assertion.

**Table 2:** Deep learning and neural networks

Researcher	Deep learning architecture	Feature types	Dataset	*F*1-score
Kabir et al. [58]	BERT, DistilBERT	BERT	DEEPTWEET [58]	
Ansari et al. [59]	LSTM with Attention	GLoVE, SenticNet	Reddit, CLPsych 2015, eRisk Dataset	0.77
Wani et al. [60]	CNN, LSTM	Word2Vec, TF-IDF	Wani et al. [60]	0.99
Nemesure et al. [61]	Stacked ensemble	Electronic health records; demographic and medical	Nemesure et al. [61]	–
Zogan et al. [62]	CNN, BiGRU	BERT	Shen et al. [42]	0.91
Wan et al. [63]	Hybrid EEGNet	Resting state EEG	Wan et al. [63]	0.95
Ray et al. [37]	BiLSTM	Audio, text and visual	DIAC [56]	–
Rosa et al. [53]	CNN, BiLSTM and RNN with SoftMax	–	Rosa et al. [53]	0.92
Tadesse et al. [32]	MLP	LIWC, LDA and Bigram	Pirina and Çöltekin [44]	0.91
Tasnim and Stroulia [36]	DNN	Audio	AVEC ’17 [64]	0.61
Alhanai et al. [34]	LSTM	Audio and text	DIAC [56]	0.77
Cong et al. [49]	XGBoost and attentional-BiLSTM	–	Yates et al. [55]	0.60
Chen et al. [57]	LSTM	–	Chen et al. [57]	–
Yang et al. [38]	Deep CNN and DNN	Audio and video	AVEC ’17 [64]	–

Table 2 provides an overview of the surveyed depression detection systems which deploy deep learning models. From this table we see a heavy reliance on text data. Recently, we observe a trend away from hand-crafted features towards complex neural word embedding models such as those seen in [59, 58, 62]. This mirrors a pattern seen in the data science field in general with powerful text embedding models becoming the current state of the art. Future research should combine interdisciplinary teams to ensure researchers are using the current leading data science techniques. The utility of these deep learning systems for the recognition of depression is quickly growing, however, to date fewer examples exist of systems that model depression treatment effect. While sophisticated deep learning networks are rapidly being utilised in research the lack of transparency of these deep neural networks comes with several limitations for their use in practice. Deep learning systems although promising in their detection are unable to justify or explain why they classify a study participant a certain way. As such, [54] argue so-called ’black box’ models should not be used in high stakes fields including healthcare, when a model is not human interpretable.

### Uncovering new diagnostic categories with unsupervised learning and data-driven informatics

Current systems of diagnosis in psychiatry rely on diagnostic labels constructed through research rather than objective measurements of disorder [4]. The problems associated with the diagnosis of mental health conditions are widely acknowledged in the literature. An observed flaw of the diagnosis of mental health conditions is the subjectivity on which it relies. Furthermore, the categorical descriptions of psychopathology ignores heterogeneity of within group variation for specific conditions. For example, Fried and Nesse [65] identified 1030 unique symptom profiles amongst 3703 patients diagnosed with clinical depression as part of the Sequenced Treatment Alternatives to Relieve Depression ($\text {STAR}^{\star }$D) trial. Fried and Nesse [65] go on to conclude “dissatisfaction with the diagnostic criteria of major depressive disorder might be reduced by acknowledging that it is not one coherent condition with a single cause” [65, p. 100].

Categorical diagnosis systems treat conditions as binary entities. Under a categorical approach disease entities or either present or absent [66]. Past research [67, 68] has sought to use neuroimaging to delineate between individuals suffering depression and healthy controls. For example, Yang et al. [68] used fMRI to compare differences in resting state activations, identifying reduced activity in the left dorsolateral prefrontal cortex when compared to prefrontal cortex. More recently, Artificial intelligence has the potential to identify sub groups within disease populations through pattern recognition. This pattern recognition can be referred to as unsupervised learning. In contrast to the supervised tasks surveyed so far, unsupervised algorithms are used to “identify inherent groupings within the unlabeled data” [69, p. 5]. Thus, unsupervised algorithms can be used to identify groupings that transcend existing diagnostic labels [70]. Exemplifying the possibility of new diagnostic criteria, Drysdale et al. [11] utilised hierarchical clustering, a type of unsupervised learning to identify four sub types of depression. Their method, grouped patients based on fMRI connectivity measures. Further exploration showed these sub types could be used to predict treatment response to rTMS. Of note the machine learning classifier was better able to predict treatment response than a model built using symptoms alone [11]. These results offer support for that position that depression may not be one single disease entity but in fact made up of multiple different conditions. More recently, Kuai et al. [71] explored a brain computing approach to construct and evaluate prediction models using different brain states. Kuai et al. [71] argue a brain mapping approach to understanding mental health offers strengths over existing strategies as it allows for hypothesis testing to validate causal results. Future work using brain computing may in fact be used to verify differences in the underlying brain structures of people diagnosed with the same condition.

This section has raised the possibility of either distinct subtypes of depression, or in fact several different underlying conditions distinct from depression. What is significant from the patients perspective is these different depression variants vary in their response to treatment. As such, the use of data to support treatment decisions in mental health has been an area of significant research. As research for personalised medicine has increased so to has work exploring the ways in which psychiatric treatments can be tailored to the individual. One emerging area of interest is the use of machine learning algorithms to predict a patient’s response to treatment prior to intervention.

## Learning systems to predict depression treatment response

Patterns of response to treatments for mental health conditions are often inconsistent. Conventional research aims to find interventions which are successful at the group level [4]. However, as highlighted above, recent research is now uncovering significant heterogeneity of symptoms among patients classified under the same diagnostic label. As such, diagnosis alone are not sufficient to inform treatments [70]. The heterogeneity of categorical diagnostic systems is reflected in the inconsistent response to treatment interventions for patients diagnosed with the same condition. Major depressive disorder provides an example of the difficulties in prescribing treatments and the inconsistency in treatment response and remission rates.

Estimates of remission rates to antidepressant treatments vary from 25 to 33% of patients achieving remission after their first course of treatment [15, 72–74]. However, this does not mean that patients do not go on to achieve remission of their disorder. Some estimates suggest 67% of patients go on to achieve remission after trials of multiple antidepressant treatments [15]. Given this, a preferred method for assigning treatments would be to maximise the likelihood of success. However, currently no standardised way exists of prescribing treatments with clinicians relying on a trial and error approach to find the best [14, 15].

A more desirable option would be to identify likely responders to an intervention prior to treatment. Under this approach, treatments can be targeted to the individual patients who are most likely to derive benefit [4]. This is the aim of precision psychiatry. Precision psychiatry supported by artificial intelligence would allow clinicians to move beyond diagnostic categories and make room for the individual variability of care [70]. Tailoring treatments to the individual has several benefits. If it is possible to predict whether a patient will respond to treatment before commencing the therapeutic intervention. Hence reducing the time spent pursuing likely ineffective treatments. Additionally, time saved reduces both the financial and psychological burden on patients and health care systems [14, 75].

### rTMS response prediction

Repetitive transcranial magnetic stimulation (rTMS) is an evidenced based treatment for depression. However, despite a demonstrated clinical benefit when compared to a control [76] for some patients rTMS is ineffective. Berlim et al. [76] in their meta analysis report a response rate to rTMS treatment of $\approx 30\%$ and remission rate of $\approx 19\%$. Similarly, Fitzgerald et al. [77] in their pooled sample review observed a response rate of $\approx 46\%$ and remission rate of $\approx 30\%$. According to Koutsouleris et al. [78] the variability of response to rTMS is seen as one of the main barriers to the widespread adaptation of the treatment modality. This section provides an overview of the data science techniques used to delineate rTMS treatment responders from non-responders. Focusing on systems which make predictions on treatment response at the level of individual patients. These treatment response prediction systems employ supervised learning techniques and utilise several types of predictor variables such as neuroimaging (MRI, EEG, fMRI), genetic, phenomenological or a combination of several variable types [79].

The works by Fitzgerald et al. [77] highlights a distinctly bimodal pattern of response to rTMS treatment. This pattern of response is distinguished by patients who respond to the rTMS treatment, and those who see little benefit. Using traditional inferential statistical techniques [77] note no variable alone could delineate between responders and non-responders. This limitation of traditional statistics highlights one strength of artificial intelligence and machine learning approaches. Advanced techniques have the ability to combine and make treatment recommendations based on multiple variables. As such, in situations where one variable alone cannot distinguish between a responder and non-responder, combinations of variables may have that power. Additionally, these advanced techniques allow for the combination of data from multiple sources. More recently, researchers [11, 14, 75, 78, 80–83] have utilised more sophisticated machine learning techniques to distinguish rTMS responders from non-responders. The works summarised in Table 3, combine physiological measurements such as electroencephalogram (EEG) [14, 75, 80–82] and fMRI [11, 83]. Table 4 provides a brief overview of the common EEG features input into the models described in this survey.

**Table 3:** rTMS treatment response prediction

Author	Condition	Features	Algorithm
Chen et al. [84]	Depression	Resting state MRI	SVM regression
Hopman et al. [85]	Depression	Resting state fMRI	Linear SVM
Bailey et al. [81]	Depression	EEG and MADRS	Linear SVM
Fan et al. [83]	Depression	Resting state fMRI	Hierarchical regression
Hasanzadeh et al. [14]	Depression	EEG	K-NN
Zandvakili et al. [75]	Depression and post-traumatic stress disorder	EEG	Lasso regression and SVM
Bailey et al. [80]	Depression	EEG	Linear SVM
Koutsouleris et al. [78]	Schizophrenia	–	Linear SVM
Drysdale et al. [11]	Depression	fMRI	Hierarchical clustering and SVM
Rostami et al. [86]	Unipolar and bipolar depression	Clinical and demographic	Binary logistic regression
Erguzel et al. [82]	Depression	EEG	Artificial neural network

Noting the link between working memory and depression (for example, [87]), Bailey et al. [80] explored the predictive power of working memory related EEG measurements. Models were built combining Montgomery Åsberg Depression Rating Scale [88, MADRS] scores, performance on a working memory test, reaction times and EEG measurements. EEG measurements included connectivity, power, and theta gamma coupling measures. Where connectivity was calculated using weighted Phase Lag Index (wPLI; [89]).

**Table 4:** EEG feature summary

Feature	Description
Cordance	The sum of *z*-transformed absolute and relative power for a frequency band [90]
Coherence	Coherence is a measure of correlation between signals [91, 92]. Contextualised, coherence is operationalised as a measure of functional connectivity between brain regions [75].
Power	A measure of the activity in a frequency band [92]
Theta gamma coupling	Research [93] has shown a relationship between theta gamma coupling and deficits in working memory
Weighted Lag Phase Index (wPLI; [89])	A measure of functional connectivity

Exploring the relationship between connectivity and rTMS response, Chen et al. [84] investigated the role of connectivity features collected using MRI. In their study, Chen et al. [84] report using functional connectivity maps as features as inputs to their SVM regression analysis. Recently, Hopman et al. [85] deployed a linear SVM using features collected via fMRI, such as connectivity features between the subgenual anterior cingulate cortex, lateral occipital cortex, superior parietal lobule, frontal pole and central opercular cortex. During fivefold cross-validation, the authors present a training accuracy of $\approx 97\%$ however, on a held out test set, model performance drops to an average $\approx 87\%$ with a 95% confidence interval from 100% to roughly 70% accuracy. Similarly, a SVM model of 30 features the [80] report an *F*1-score of 0.93 and a balanced accuracy of 91%. These metrics were the mean results of a robust internal validation scheme of 200,000 iterations of fivefold cross-validation. Building upon these initial findings [81] explored the utilised linear SVM with resting EEG features collected prior to treatment and after 1 week of treatment to predict rTMS treatment response for depression. Built using 54 features the research utilised 5000 trials of fivefold cross-validation to achieve a balanced prediction accuracy of 86.6%. The 54 features combined measures collected from MADRS questionnaire and quantitative EEG signals Alpha Power, Theta Power, Alpha Connectivity, Theta connectivity, Theta Cordance and Individualised Alpha Peak frequency. Building upon [81, 75] used machine learning to predict response to rTMS of depression sufferers with comorbid post-traumatic stress disorder (PTSD). However, in contrast to Bailey et al. [81], Zandvakili et al. [75] utilised lasso regression to model treatment prediction. Alpha EEG signal coherence was used to build the lasso prediction model. Coherence is a measure of correlation between signals [91, 92]. Contextualised, coherence is operationalised as a measure of functional connectivity between brain regions [75]. Utilising a regression model the model outputs predicted percentage reductions in scores on the Post-Traumatic Stress Disorder Checklist-5 (PCL-5; [94]) and Inventory of Depressive Symptomatology-Self-Report (IDS-SR; [95, 96]). Reductions of greater than 50% are classified as a clinical response. Continuous predictions of questionnaire score reduction are then converted to classifications. For example, a model that predicts a 60% reduction in IDS-SR for an actual reduction of 65% is the correct. While Zandvakili et al. [75] report an impressive AUC of 0.83 utilising Alpha coherence to predict IDS-SR response and AUC of 0.69 for PCL-5 response classification. These results must be interpreted in the context of high sensitivity (approx. 100%) and low specificity (approx. 50%) suggesting a large number of false positives.

Continuing with the use of pretreatment EEG features [14] sought to predict treatment response to rTMS. Where response was defined as a reduction of Hamilton Rating Scale for Depression (HRSD; [97]) or Beck Depression Inventory (BDI; [98]) by over 50%. Their sample included 46 patients with a balanced sample of responders and non-responders. The model utilised K-NN built on EEG features with the best single feature model built using the Power of beta. This model achieved a classification accuracy of 91.3% when using leave one out cross-validation. The best performing of the multi-feature models included the Power measurements of all bands (Delta, Theta, Alpha, Beta) accuracy remained at the level as the model built using only the power of Beta. However, the model utilising all power features did differ in terms of specificity and sensitivity. Hasanzadeh et al. [14] claim their system built using only pretreatment EEG features offers a better alternative to systems requiring multiple measurements.

To our knowledge [82] provides the only example of a deep learning algorithm for the prediction of rTMS responders. Erguzel et al. [82] explored the possibility of quantitative EEG to predict treatment response using an artificial neural network. The main predictive model utilised Quantitative EEG (QEEG) cordance as the main predictive feature, this is consistent with Bailey et al. [81] who offer some support for the use of cordance as an input feature. Further evidence [99, 100] suggests theta cordance for the discrimination between treatment responders and non-responders. The majority of surveyed papers relying on EEG use hand-crafted features consisting of existing signal processing techniques. However, more recently [63], showed through a novel deep learning CNN, EEG data can be processed directly by a deep learning architecture. This provides an opportunity for future researchers to streamline the data pipeline by inputting EEG data directly into networks.

The literature so far has highlighted the value of rTMS treatment for at a minimum a subset of the population experiencing depression. Additionally, emerging evidence exists to support the use of rTMS for the treatment of schizophrenia [101, 102]. Koutsouleris et al. [78] utilised linear SVM to predict treatment response for schizophrenia to rTMS treatment. Utilising structural MRI they utilised principal component analysis to reduce image features to approximately 25 principal components. According to Koutsouleris et al. [78] response was defined using the positive and negative syndrome scale (PANSS; [103]). In contrast to depression, schizophrenia is characterised by both positive symptoms including hallucinations and delusions as well as negative symptoms such as social withdrawal [104]. As such, response to treatments for schizophrenia is defined as a greater than 20% increase in the positive symptoms sub-scale (PANSS-PS) or greater than 20% increase in the negative symptom sub-scale (PANSS-NS). Hence, response to treatment is classified in terms of response for positive symptoms or negative symptoms. In the active treatment condition a cross validated model produced a balanced accuracy of 85% between responders and non-responders. Consistent with expectation and findings observed by Tian et al. [105] when utilising a leave-one-site-out validation protocol was utilised balanced accuracy dropped to 71%. Koutsouleris et al. [78] provides evidence for machine learning algorithms utility irrespective of condition. With enough data, advanced computing techniques have the potential to support improvements across multiple conditions in psychiatry.

To that end, prediction of responders at the single patient level has become of interest to the research community. The surveyed papers show EEG features to be the most common neuroimaging feature [14, 75, 80−82], with a recent trend towards fMRI and MRI features [83–85]. EEG measurements of interest include connectivity, measured using coherence or wPLI, along with power and cordance. Additional features include depression rating surveys such as MADRS [81]. These observations are consistent with Lee et al. [79] who explored the use of machine learning algorithms to predict treatment outcomes for patients with either depression or bipolar depression. In the current work SVM was the most widely used algorithm to delineate between treatment responders and non-responders of rTMS treatments. Several studies report exceptional predictive performance (for example, [80]) for their models, however, the studies surveyed rely almost exclusively on cross-validation, an internal validation strategy. Of note [14, 78] included some pseudo-external validation in the form of a leave one group out validation. In their multi-site sample, validation involved holding one site out from training for model evaluation. Interestingly, performance of this model dropped significantly when tested on a site not included in the training set. Future opportunities exist for the streamlining of techniques to preprocess data such as EEG, MRI and fMRI for input into deep learning models. Future work may see networks which automate this preprocessing reducing the need for hand-crafted features.

### Pharmacological intervention response prediction

Currently, robust biomarkers or objective measurements of psychiatric conditions do not exist. However, several studies have identified neuroimaging techniques as “candidates of prognostic biomarkers in major depression disorder” [72, p. 2]. Seminal work by Khodayari-Rostamabad et al. [15] provides an early example of treatment response prediction for antidepressants. Their system utilised pretreatment EEG features combined with a mixed feature analysis [106]-based classifier to predict treatment response prediction. More recently, Jaworska et al. [72] explored the efficacy of several machine learning classifiers for the prediction of treatment response of antidepressants. The work explored, random forests, Adaboost, SVM, classification and regression trees (CART) and the multilayer perceptron (MLP). The best performing model reported by Jaworska et al. [72] was a random forest classifier which combined 117 features from a variety of sources including eLORETA, EEG and clinical features. The model recorded an *F*1-score of 0.901. Despite this impressive performance, models built with large numbers of features are vulnerable to overfitting [107]. Given the problem of overfitting, the more suitable model presented by Jaworska et al. [72] is built using twelve predictive features selected based using extremely randomised trees. This method ranks the predictive power of features using the average impurity score. Of models built using only twelve features, [72] report random forest to have the best prediction performance with an *F*1-score of 0.827 slightly outperforming Adaboost with an *F*1-score of 0.815. Similar to the findings of Drysdale et al. [11], Jaworska et al. [72] assert models built on features incorporating imaging techniques outperformed models built solely on clinical or demographic data. This assertion suggests models neuroimaging techniques to be a more reliable measure of psychiatric health.

**Table 5:** Pharmacological treatment response prediction

Author	Features	Algorithm	Validation
Jaworska et al. [72]	EEG and eLORETA	Random forests	Tenfold cross-validation
Browning et al. [108]	Initial QIDS-R and face-based emotional recognition task (FERT)	Linear SVM	External validation on unseen data
Pei et al. [109]	EEG and genetic markers	Linear SVM	Leave-one-out cross-validation
Chang et al. [16]	MRI and genetic markers	Artificial neural network	Holdout set and k-fold cross-validation for hyperparamater tuning
Tian et al. [105]	fMRI	Linear support vector machine	Leave-one-out cross-validation
Carrillo et al. [10]	Speech data	Gaussian Naive Bayes	Sevenfold cross-validation
Lin et al. [110]	Genetic markers	Multilayer feedforward neural network	10 iterations of tenfold cross-validation
Mumtaz et al. [111]	EEG	Logistic regression	100 iterations of tenfold cross-validation
Chekroud et al. [112]	Sociodemographic, questionnaires (such as HAMD), clinical information	Gradient boosting machine	10 iterations of tenfold cross-validation and externally validated on unseen data
Patel et al. [113]	Demographic and neuroimaging	Alternating decision trees	Leave-one-out cross-validation
Khodayari-Rostamabad et al. [15]	Pretreatment EEG	Mixture of factor analysis	100 iterations of leave N out cross-validation

While imaging, clinical and demographic features are the predominant features of interest, pioneering works [16, 109, 110] have included genetic features, such as single nucleotide polymorphisms (SNP). Pei et al. [109] collected SNP’s via a blood sample where the significance of each allele was determined using logistic regression. The outcome variable of interest was treatment response vs non-response. Continuing with the theme of algorithmic feature set selection, Pei et al. [109] utilised SVM recursive feature elimination. Linear SVM was used in an ensemble approach outperforming single classifiers built using the same predictor variables. This result is consistent with the literature that emphasises the strength of ensemble methods for classification tasks in supervised learning [114]. Similarly, Lin et al. [110] explored the predictive power of SNPs utilising the deep learning algorithm, multilayer feedforward neural networks (MFFN). The work explored the performance capability of the MFFN compared to logistic regression with a feature set of 16 biomarkers and six clinical features to predict both treatment response and remission. For a set of 16 features, the MFFN with up to three hidden layers outperformed logistic regression in both AUC and sensitivity, however, logistic regression achieved slightly better specificity. When the number of features was lowered to six biomarkers, similar to Jaworska et al. [72] performance declined as the number of features dropped. For 6 features, the best AUC score dropped to an AUC of 0.5597 for a single-layer MFFN with the logistic regression achieving higher specificity.

Also utilising a deep learning for the prediction of treatment response, Chang et al. [16] developed a neural network based system, the Antidepressant Response Prediction Network (ARPNet), to predict both the degree of treatment response, as a continuous variable, and whether a patient reaches clinical remission. In contrast to other studies (see [72, 109]), Chang et al. [16] define clinical remission as a greater than 50% reduction in HAM-D score; whereas [110] defined remission as a HDRS score of less than 7. These differences in definitions are significant. As the field strives for clinical use of artificial intelligence systems a standardisation of definitions would be helpful for comparing models. Despite terminology differences, Chang et al. [16] present a robust system to predict response with their model significantly outperforming other widely used classifiers such as linear regression. Similar to Pei et al. [109], Lin et al. [110], ARPnet includes genetic variables and combines this information with neuroimaging biomarkers. The system utilises elastic net feature selection with hyper parameter tuning conducted using fivefold cross-validation with a test set of 10%. Two features unique to ARPnet is the antidepressent prescription layer of the neural network and the use of ARPnet to predict the degree of treatment response, measured in terms of HAM-D score across time. This novel approach would allow psychiatrists to model the likely response of an antidepressant before prescribing it [16].

While text features were widely used for the detection of depression (see Sect. 2), the use of these features is uncommon in treatment response prediction. Carrillo et al. [10], in a unique method present text analysis as a method for predicting the treatment response to psilocybin. Given, the established relationship between psychological health and language use [115–119], Carrillo et al. [10] first show that a Gaussian Naive Bayes classifier could distinguish between individuals suffering from depression, and healthy controls. Their model was built using features constructed by sentiment analysis collected via interview. Additionally, this Gaussian system able to distinguish responders from non-responders at a level of significance when compared to permutation testing. However, this research is significantly limited by the small sample size of only 17 study participants comprising 7 responders and 10 non-responders.

So far this section has explored a variety of data sources used as features for systems that predict treatment response. With the most common physiological feature being EEG. An additional and emerging data type is the use of fMRI neuroimaging [11, 83, 105]. Tian et al. [105] explored resting fMRI features as predictors of escitalopram response in patients suffering depression. The work explored the predictive power of fMRI features across three sites. Using data of 34 patients from Nanjing Brain Hospital across a 7-year period [105] used an SVM classifier to deliver an optimal accuracy of 79.41%. Using permutation test as comparison the authors [105] conclude this result to be significant at the *p* < 0.001 level. Using the minimum redundancy maximum relevancy the authors identified 7–8 features which combined to produce the optimal classifier. Similar to Hasanzadeh et al. [14], Koutsouleris et al. [78], as Tian et al. [105] was a multi-site trial, a leave one group/site out analysis was used as a validation technique. Using one site as the hold out set for more thorough validation which tests model generalisation. For Tian et al. [105] a leave one group out analysis showed performance decrease. This leave one group out protocol achieved accuracy of between 69 and 71% compared to the 79.41% when data were trained and tested at a single site. This performance drop highlights the common limitation of machine learning, model generalisation to unseen data. Similar performance decline is observed by Browning et al. [108] who provide one of few examples of external validation on an independent dataset. Exploring the possibility of baseline Quick Inventory of Depression Severity (QUIDS; [120]) and the face-based emotion recognition task (FERT). Browning et al. [108] observed performance decline from approximately 80% accuracy to 60% accuracy on the independent dataset. Similarly, Chekroud et al. [112] using gradient boosting machines achieved an accuracy score 64.6% during cross-validation compared to an accuracy of 59.6% on an external data a performance drop not in the magnitude of Browning et al. [108]. The difference in relative performance drop could be due to the low accuracy reported in the internal validation stage by Chekroud et al. [112]. Performance comparisons between Browning et al. [108] and Chekroud et al. [112] are further complicated by their different target variables. Browning et al. [108] sought to identify patients who achieved a response to treatment, defined by a greater than 50% reduction in QIDS-SR, in contrast, Chekroud et al. [112] sort to identify clinical remission defined by the QIDS-SR as a final score less than or equal to five.

**Table 6:** Pharmacological treatment response sample summary

Author	Sample size	Definition of response
Jaworska et al. [72]	51	$>50\%$ reduction in MADRS score
Pei et al. [109]	98	$>50\%$ reduction in HDRS 6
Lin et al. [110]	421	–
Chang et al. [16]	121	Remission defined as $>50\%$ reduction in HAM-D
Carrillo et al. [10]	17	$>50\%$ reduction in QIDS
Mumtaz et al. [111]	34	$>50\%$ reduction in BDI-II
Khodayari-Rostamabad et al. [15]	22	$>30\%$ reduction in HAM-D

Several algorithms have been trialled for the prediction of treatment response to pharmacological treatments of depression. A summary of these techniques can be found in Table 5. These algorithms include deep learning techniques such as MFFN [72] and customised neural net-based systems such as those in Chang et al. [16]. Other commonly utilised algorithms include Linear SVM [109, 105], tree-based methods [72, 113] and logistic regression [111].

While the majority of studies discussed in this section report impressive results, they are significantly limited by small samples (see Table 6) and lack of external validation. Commonly, internal validation techniques such as k-fold cross-validation and leave-one-out cross-validation. And others [110, 111] employed repeated cross-validation, the most robust form of internal validation [121]. We observed significant performance drops when data were spread across multiple sites or models tested on independent data. This performance decline highlights the issue of generalisation in machine learning, one of the key barriers to clinical adoption of these techniques [5, 122].

We also note the recent shift towards more sophisticated deep learning techniques, with Tian et al. [105] claiming their MFFN to outperform a logistic regression, [16] reporting their neural net-based system to outperform common strategies such as SVM and random forests. The majority of response prediction studies agreed to a common definition of response as a greater than 50% reduction in score from a psychometric questionnaire used to asses depression severity, with instrument of choice varying across samples. Notably, only Chang et al. [16] differed in their definition responder, defining clinical remission as a 50% reduction in HAM-D score.

As artificial intelligence becomes more prevalent in medicine and psychiatry a more standardised framework is required for the testing and validation of deep learning models. Differences in definitions between models make comparison between systems more difficult. As such regulators and the research community should endeavour to standardise definitions; This standardisation would first make the regulation of artificial intelligence systems easier and secondly make communication of model performance more transparent.

## Discussion: challenges and opportunities

Advances in deep learning, machine learning and natural language processing are slowly being applied to the field of precision psychiatry. This paper serves as a guide for psychiatrists and data science practitioners alike as to the existing state-of-the-art techniques and the open problems which require further work.

Supporting a shift towards precision psychiatry artificial intelligence provides the opportunity for treatment response prediction. Treatment response prediction provides empirical evidence for the likely effect of an intervention. Currently, clinicians rely on trial and error to find the best antidepressant for a patient [4, 14, 15]. As such, treatment response prediction offers a shift from trial and error treatment prescription to evidence-based treatment recommendations supported by data. The surveyed works explore two categories: single patient response prediction for rTMS and pharmacological interventions. These systems utilise any of neuroimaging, demographic and clinical features [79]. Jaworska et al. [72] observed neuroimaging features outperformed clinical and demographic features. This is consistent with Drysdale et al. [11] reports “clinical symptoms alone were not strong predictors of rTMS treatment responsiveness at an individual level” [11, p. 8]. Systems built using neuroimaging techniques consistently demonstrated the ability to delineate between treatment responders and non-responders for both rTMS and drug-based treatments. However, for these systems to be adopted in a clinical setting several limitations must be addressed.

### Challenges and limitations

Through our survey of the literature, we identified some consistent themes for consideration by the research community. The studies reviewed so far report impressive results for the detection, diagnosis and treatment response prediction. Despite impressive results reported above, none of the works surveyed as yet have been shown to demonstrate improved treatment outcomes for patients. Given the field of personalised psychiatry is not new, with surveyed works spanning a decade. Further collaboration between mental health professionals and data scientists to ensure this research is being converted into improved patient outcomes. This section explores the limitations of existing systems which reduces the possibility of real world application.

#### Model validation: the need for external validation

Several of the surveyed studies described in previous sections report impressive power for predicting treatment response with several performing above current standards observed in practice. However, several issues exist in moving these research systems to clinical practice. Of the papers reviewed above the most obvious limitation, or barrier to implementation is the issue of model validation.

Of the surveyed articles two studies include multiple sites [78, 105] and two test their models on independent data [108, 112]. Rigorous validation is crucial if machine learning systems are to effectively transition to industry use [122]. The majority of papers cited above use some form of internal validation such as k-fold cross-validation. Widely cited work by Harrell Jr [121] provides a hierarchy of validation techniques used to predict model performance on new data. Using this hierarchy validation techniques range in effectiveness from only reporting the best performing iteration of model performance, to the most powerful validation technique, external validation by an independent research team on new data. Harrell Jr [121] asserts the strongest of internal validation techniques is repeated iterations of k-fold cross-validation. Model validation is of significant importance in the transition of predictive models. Fröhlich et al. [5] notes the path to implementation for predicative artificial intelligence models must include robust internal validation, external validation on independent data and empirical validation as part of a clinical trial.

These views are supported by Browning et al. [108] who contend randomised control trials are necessary to validate model performance to a level that would justify clinical adoption. Of the papers surveyed to date few tested their models on independent data and none included randomised control trials of their systems. With the lack of publicly accessible data for depression, external validation of model performance is challenging. Open datasets would enable researchers to build their models on one dataset and compare performance across samples. This realisation is already being realised by datasets such as ADNI, providing an established research pipeline for the study of Alzheimer’s. Providing researchers with datasets for external validation.

#### Small sample sizes and greater data access

The issue of access to data and sample sizes provides a brief overview of progress in the respective dimensions covered in this review. Data relating to depression detection are widely available compared to data for treatment response prediction. For example, social media text, DIAC [56] and AVEC [64] are widely accessible. Access to data provides computer scientists and researchers the opportunity to compare their systems on the same datasets. In contrast, researchers exploring treatment response prediction at the single patient level are limited by small samples and challenges accessing data. A centralised cloud-based repository of mental health data as proposed by Chen et al. [123] offers one potential solution, however, would be require significant infrastructure to implement.

Treatment response prediction relies more heavily on neuroimaging data. Labelled examples for treatment response prediction are far less available with the surveyed articles relying on small samples. Table 6 provides an overview of the sample sizes used to generate the results discussed in this paper. Consistent with trends identified in Arbabshirani et al. [124], with the exception of [110] the majority of studies surveyed have samples under 150. Arbabshirani et al. [124] assert it is difficult to generalise results from small samples to the broader patient population. Furthermore, it is likely small samples overstate the predictive power of a system [125]. Button et al. [126] assert low statistical power as a result of small sample sizes is a problem of endemic proportions within the field of neuroscience. Combined, with observed publication bias of artificial intelligence systems [125] it is likely the published literature provides only a theoretical upper limit of the current effectiveness of artificial intelligence systems for precision psychiatry. Furthermore, small sample sizes do increase the probability of overfitting [4], leaving researchers to overstate the performance of their model.

For the continued growth of personalised psychiatry research larger datasets become more accessible. The dearth of open datasets is especially true for the study of depression. With the benefits of open data sharing is exemplified by the success garnered from the Alzheimer’s Disease Neuroimaging Initiative. Recently, Birkenbihl et al. [122] report the ADNI dataset has now been referenced more than 1300 times. To date there is no equivalent data repository for conditions such as depression. Possible large cloud based solution such as that proposed by Chen et al. [123] may pave the way forward, however, further work is required.

### Future trends and opportunities

The last decade of research has seen rapid advancements in the technologies being used to support mental health care. For the detection and diagnosis of depression we observe a trend away from machine learning algorithms to sophisticated deep learning architectures. Similarly, text classification is moving away from traditional text mining features such as *n*-grams and bag-of-words to more sophisticated transformer-based embeddings such as BERT. However, the transition to deep learning architectures is less evident in treatment response prediction. Despite using quantitative data like EEG, fMRI or MRI, this field is relying on existing technologies such as SVM. Few methods exist where raw neuroimaging data, such as EEG is passed directly to Deep Learning Algorithms. Thus an opportunity exists for the use of deep learning methods to learn feature representations for the treatment response prediction and streamline data preparation.

#### Causal artificial intelligence

Existing trends in this survey show a move from hypothesis testing, to pattern recognition using artificial intelligence techniques. However, predictive techniques do not establish causality as hypothesis and randomised control trials did. While some confuse pattern recognition for causality, Sgaier et al. [127] asserts “Relying solely on predictive models of AI in areas as diverse as health care, justice, and agriculture risks devastating consequences when correlations are mistaken for causation.”

Establishing causation using artificial intelligence would be a significant breakthrough in depression research and precision psychiatry alike. In some medical fields we are starting to see early attempts at establishing causality with the use of deep learning. Wang et al. [128] show their model DeepCausality was able to identify 20 causal factors for identifying drug induced liver disease from electronic health records. Furthermore, advances in brain mapping such as the strategies shown in Kuai et al. [71] may allow for the establishment of causal relationships between changes in brain activity and depression severity

#### New technologies and automating data pipelines

Recent advances in text embeddings such as BERT, GloVe or Word2Vec are more often being utilised by practitioners to prepare text for depression detection. The use of these transformer-based word embeddings have led to more streamlined data pipelines. Further opportunities exist for data scientists to develop new techniques to process neuroimaging data directly such as the approach proposed by Wan et al. [63]. CNNs are well equipped to handle sequence data and feature work may allow for networks equipped to handle neuroimaging data without prepossessing.

To date, the detection and diagnosis of mental health conditions relies on self-report or clinician-administered questionnaires. Currently, objective biomarkers of psychopathology do not exist [11]. Given this challenge, significant research has explored the possibility of depression detection using text, audio and visual. Currently, evidence [37] suggests the content of speech is the best predictor when compared to audio and visual to delineate between people who are healthy and individuals suffering mental health conditions. Systems designed for depression detection utilise a variety of techniques progressing from elementary machine learning methods to more sophisticated techniques such as deep learning algorithms. Depression detection is the most widely researched area explored within the scope of this survey. This advancement has been driven by the access to significant bodies of text and publicly accessible datasets such as DIAC [56] and AVEC [64].

#### Uncertainty quantification

As the field strives for clinical implementation of the artificial intelligence systems surveyed further work is required to capture the uncertainty associated with model building. This includes the two types of uncertainty, data uncertainty (aleatoric uncertainty), and epistemic uncertainty, (model uncertainty). The aleatoric uncertainty can be seen in the variations in depression detection system performance depending on how ground truth labels were collected. We noted performance drop off when self-report measures were used as ground truth labels. The use of self-report measures encompasses some inherent uncertainty which existing methods fail to capture. Additionally, if these models are to become prevalent in their use in informing treatment decisions, these models must be able to express their prediction confidence, which currently is not included in model outputs. Bayesian Neural Networks are an emerging technology to encompass both data uncertainty and express prediction confidence. Further to this, more work is required to ensure as models become more complex effort is made to understand the inner workings of these models. Some concerns exist regarding the lack of transparency in how deep learning models make their predictions. These concerns have led some [54] to argue against the use of deep learning models for important health-related decisions. Accurate predictive models which are interpretable are of significant interest to the research community.

## Conclusions

Much excitement surrounds the potential for artificial intelligence and machine learning to revolutionise psychiatry. This paper provides an overview of the techniques and methodologies available to researchers for the detection, diagnosis and treatment of depression. Whilst every endeavour has been made to ensure the completeness of this survey paper given the speed of progress within the data science community we cannot guarantee all papers within the literature have been included. However, this paper aims to provide an up-to-date assessment of the current position of artificial intelligence’s use in the field of psychiatry.

The last decade of research has seen rapid advancements in the technologies being used to support mental health care. For the detection and diagnosis of depression we observe a trend away from machine learning algorithms to sophisticated deep learning architectures. Similarly, text classification is moving away from traditional text mining features such as *n*-grams and bag-of-words to more sophisticated transformer-based embeddings such as BERT. However, the transition to deep learning architectures is less evident in treatment response prediction. Despite using quantitative data like EEG, fMRI or MRI, this field is relying on existing technologies such as SVM. Few methods exist where raw neuroimaging data, such as EEG is passed directly to deep learning algorithms. Thus an opportunity exists for the use of deep learning methods to learn feature representations directly and streamline the treatment response prediction process.

Current limitations of treatment response systems include small sample sizes and model validation. The small samples observed in the treatment response prediction systems described in Sect. 3 make it difficult to generalise findings to the broader population [124]. Additionally, small sample sizes increase the likelihood of model overfitting [4]. Larger, more publicly accessible datasets such as the data pipelines that are well established for the study of Alzheimer’s disease (see [122]) would address this issue. Further barriers to the widespread adoption of these systems is the issue of model validation. As noted by Fröhlich et al. [5] the path to implementation for predicative artificial intelligence models includes robust internal validation, external validation and empirical validation as part of a clinical trial. Of the works included within the scope of this review the majority includes only internal validation, falling well below the standard for implementation. To advance the field of personalised psychiatry to the clinic, future work should seek larger datasets and explore empirical validation in the form of randomised control trials. We suggest greater collaboration between healthcare professionals and artificial intelligence researchers may speed up the process of adoption and ensure state-of-the-art techniques are being used to improve health outcomes.

## Data Availability

Not applicable.
